# *Cryptalyra* (Hymenoptera, Megalyridae) from Maranhão, Brazil: three new species discovered after a large collecting effort

**DOI:** 10.3897/zookeys.442.8237

**Published:** 2014-09-25

**Authors:** Ricardo Kawada, Diego N. Barbosa, Celso O. Azevedo

**Affiliations:** 1 Universidade Federal do Espírito Santo, Departamento de Ciências Biológicas, Av. Fernando Ferrari 514, Goiabeiras, 29.075-910, Vitória, ES, Brazil

**Keywords:** Apocrita, Cryptalyrini, Neotropical, taxonomy, Malaise trap

## Abstract

*Cryptalyra* is a small genus of megalyrid wasps with three described species confined to South America. Our main goal in this work is to record an increase in the known diversity, adding the three new species *Cryptalyra helenae* **sp. n.**, *Cryptalyra ichiroi* **sp. n.** and *Cryptalyra limeirai* **sp. n.**, from a single locality in Maranhão, Brazil that has been subjected to extraordinary collecting effort. We are providing a key for the species of the genus, as well as illustrations of the main structures for recognition of the new taxa.

## Introduction

Megalyridae have 49 extant species allocated in eight extant genera (Vilhelmsen et al. 2010). The information available comes primarily from a few members of *Megalyra* (Rodd 1951, Naumann 1987, Shaw 1990a) most attacking xylophagous Coleoptera.

Shaw (1987, 1988, 1990a) surveyed and revised the majority of extant Megalyridae; Shaw (1990b) summarized the classification, analyzed the phylogeny of the extant and extinct members of the family known at the time.

Shaw (1987) described three monotypic genera from the Neotropics: *Cryptalyra* Shaw from the tropical and subtropical part east of the Andes, and *Neodinapsis* Shaw and *Rigel* Shaw from Chile in the temperate Neotropics. In terms of species, the Neotropical megalyrid fauna seems to be very depauperate. However, the extant generic diversity is on a par with that of the other Southern Hemisphere regions, and, as in the Afrotropics, the poor species turnout might yet prove to be the result of lack of exploration.

*Cryptalyra* Shaw, 1987 is a small genus confined to South America (Azevedo and Tavares 2006). They are *Cryptalyra colombia* Shaw, 2003, from Colombia, *Cryptalyra plaumanni* Shaw, 1987 from Southern Brazil and *Cryptalyra depressa* Azevedo and Tavares, 2006 from Northern Brazil. All of them were described based on a single female.

While visiting the collection of Universidade Estadual do Maranhão in Caxias we found ten more specimens of *Cryptalyra*, which correspond to three new species. Thus the main goal of this contribution is to formally describe them.

## Material and methods

The material examined was provided by Francisco Limeira-de-Oliveira, curator of the Coleção Zoológica do Maranhão (CZMA), Universidade Estadual do Maranhão (UEMA), Caxias, Maranhão, Brasil.

The terminology follows Shaw (1987, 2003), Vilhelmsen et al. (2010) and the Hymenoptera Anatomy Ontology (HAO) project (Yoder et al. 2010, Seltmann et al. 2012). The nomenclature of integumental sculpture follows Harris (1979). The key to species follows Azevedo and Tavares (2006). All new species have been prospectively registered with Zoobank (Polaszek et al. 2005).

The specimens were examined under a Leica MZ80 Stereo Microscope. Images were taken with a Leica DFC 495 video camera attached to a Leica Z16 APO with a Planapo 2.0x objective. Figures were produced from stacks of images that vertically transected the specimen using Leica LAS (Leica Application Suite V4.3.0) Microsystems by Leica (Switzerland) Limited. These were combined automatically into a single image using Helicon Focus (version 6.0.18), based on Method C (Pyramid) and focus autoadjustments 1% (horizontally).

## Results

### Key to females of *Cryptalyra* modified from Azevedo and Tavares (2006)

**Table:** 

1	Propodeum with wide longitudinal depression	2
–	Propodeum without such depression (Figs 6B, 7B, 8B)	3
2	Body light chestnut brown; middle of face to vertex crest without longitudinal sulcus; median mesoscutal line as continuous sulcus; propodeum with longitudinal depression delimited by lateral carina	*Cryptalyra depressa* Azevedo & Tavares
–	Body black; middle of face to vertex crest with longitudinal sulcus (Figs 4A/B); median mesoscutal line deep, broad and scrobiculate (Fig. 6B); propodeum with longitudinal depression not delimited by lateral carina	*Cryptalyra helenae* sp. n. (Fig. 1)
3	Propodeum with posterior tubercle	*Cryptalyra plaumanni* Shaw
–	Propodeum without posterior tubercle (Figs 8, 9)	4
4	Median mesoscutal line formed by continuous fovea, unbounded and widening posteriorly (Fig. 8B); metatibia with two apical spurs (Fig. 12F)	*Cryptalyra limeirai* sp. n. (Fig. 3)
–	Median mesoscutal line formed by separated fovea (Fig. 7B); metatibia with one apical spur	5
5	Eye densely setose; frons densely foveolate; hind wing Rs vein not reaching middle of wing; lower valve with three minute apical teeth	*Cryptalyra colombia* Shaw
–	Eye sparsely setose (Figs 4C/D); frons densely coriaceous (Fig. 4C); hind wing (Fig. 10C) Rs vein almost reaching middle of wing; lower valve with five minute apical teeth	*Cryptalyra ichiroi* sp. n. (Fig. 2)

**Figure 1.**
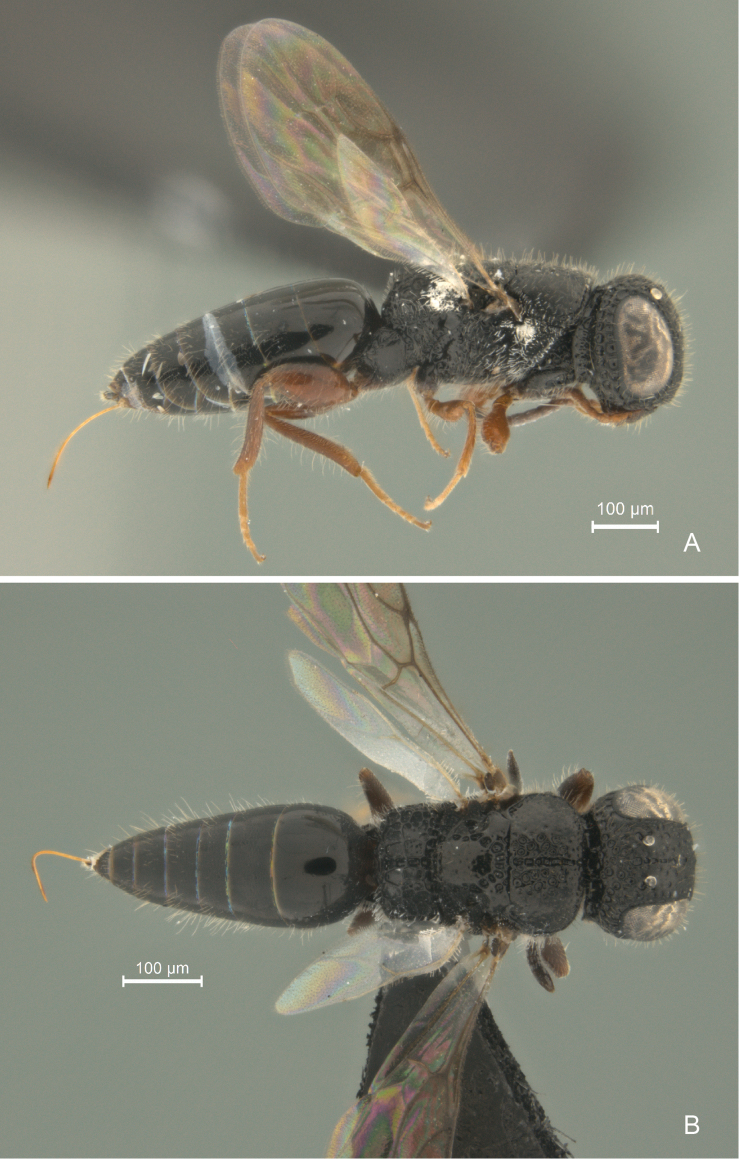
*Cryptalyra helenae* sp. n. **A** habitus in lateral view **B** habitus in dorsal view. Scales bar in microns.

**Figure 2.**
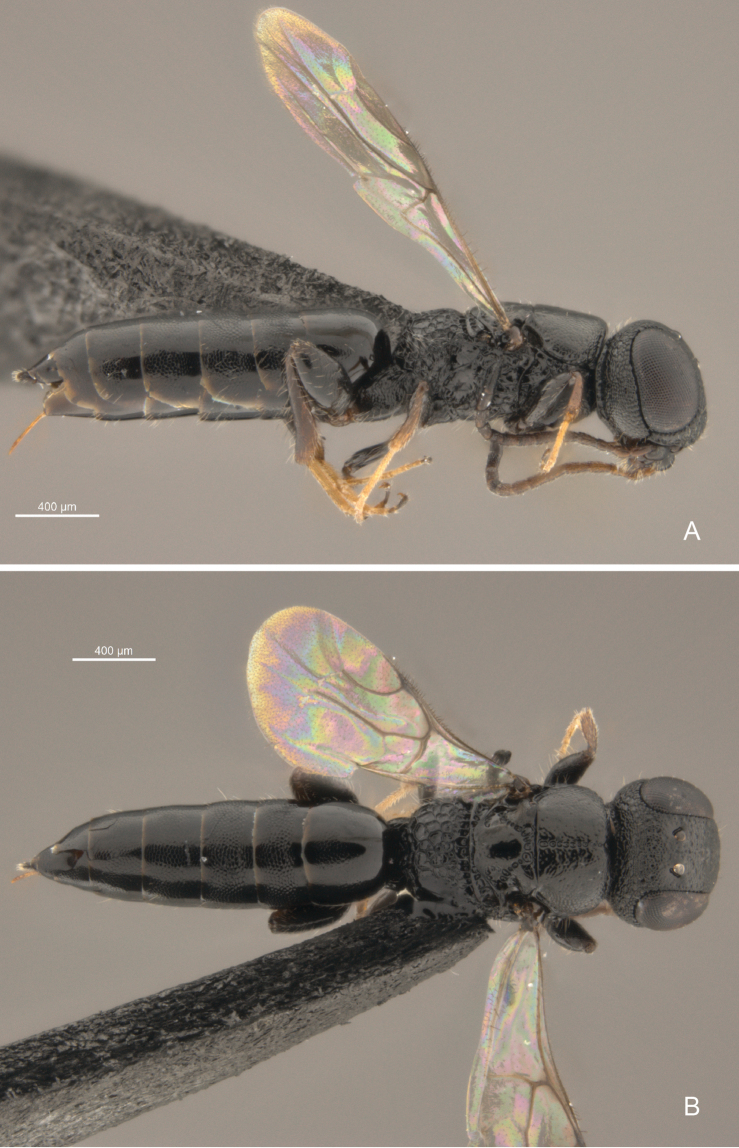
*Cryptalyra ichiroi* sp. n. **A** habitus in lateral view **B** habitus in dorsal view. Scales bar in microns.

**Figure 3.**
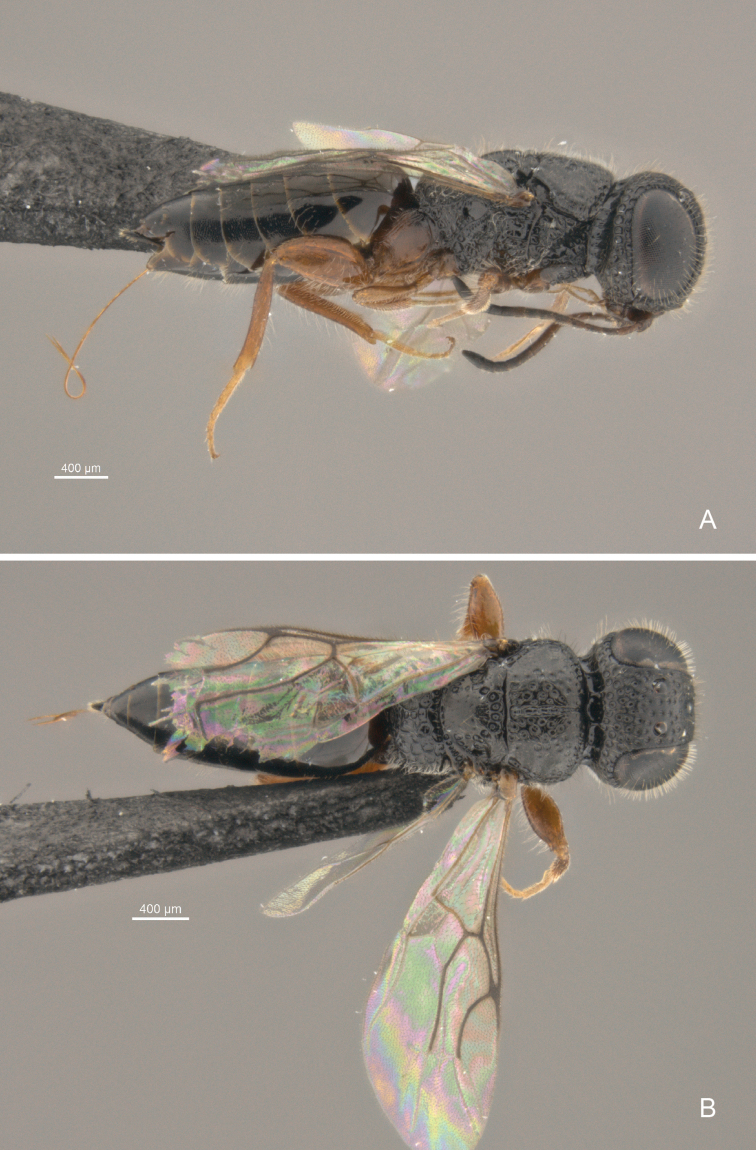
*Cryptalyra limeirai* sp. n. **A** habitus in lateral view **B** habitus in dorsal view. Scales bar in microns.

**Figure 4.**
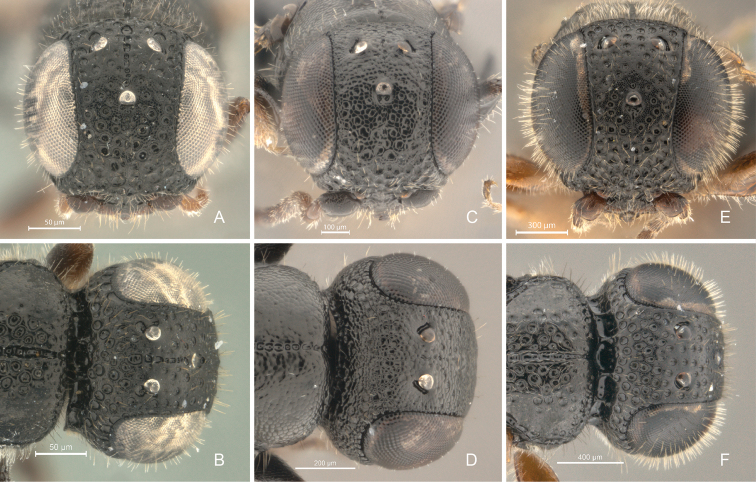
*Cryptalyra helenae* sp. n. **A** head in frontal view **B** head in dorsal view. *Cryptalyra ichiroi* sp. n. **C** head in frontal view **D** head in dorsal view. *Cryptalyra limeirai* sp. n. **E** head in frontal view **F** head in dorsal view. Scales bar in microns.

**Figure 5.**
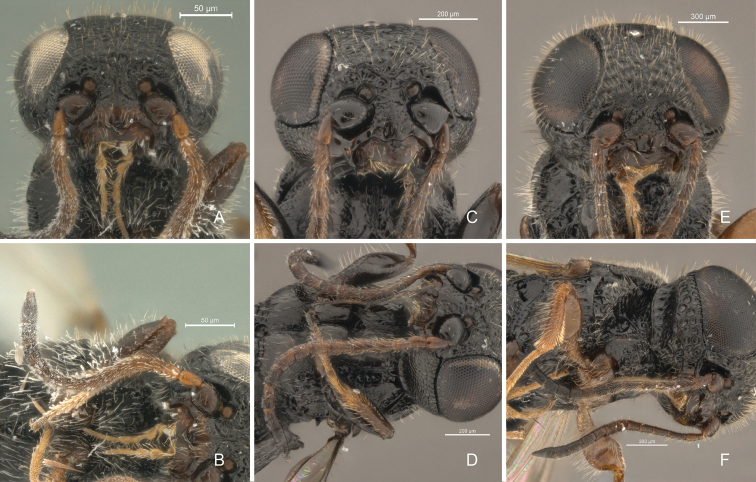
*Cryptalyra helenae* sp. n. **A** head in ventral view **B** antenna in dorsal view. *Cryptalyra ichiroi* sp. n. **C** head in ventral view **D** antenna in dorsal view. *Cryptalyra limeirai* sp. n. **E** head in ventral view **F** antenna in dorsal view. Scales bar in microns.

**Figure 6.**
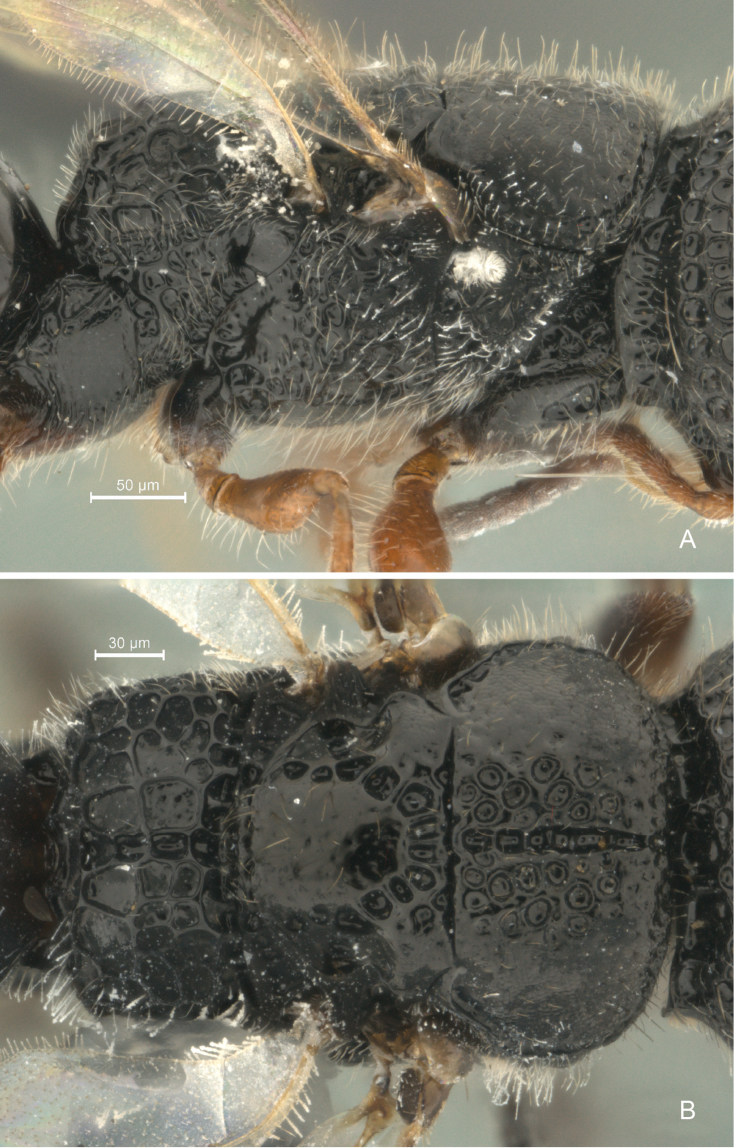
*Cryptalyra helenae* sp. n. **A** mesosoma lateral view **B** mesosoma dorsal view. Scales bar in microns.

**Figure 7.**
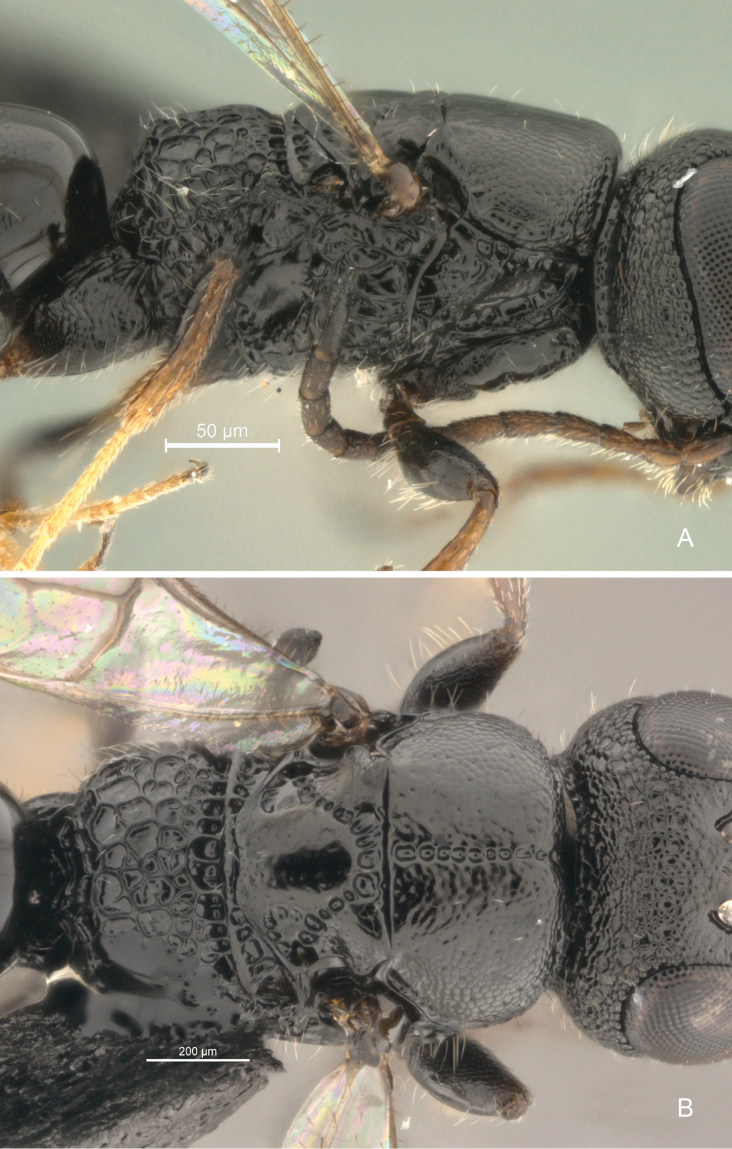
*Cryptalyra ichiroi* sp. n. **A** mesosoma lateral view **B** mesosoma dorsal view. Scales bar in microns.

**Figure 8.**
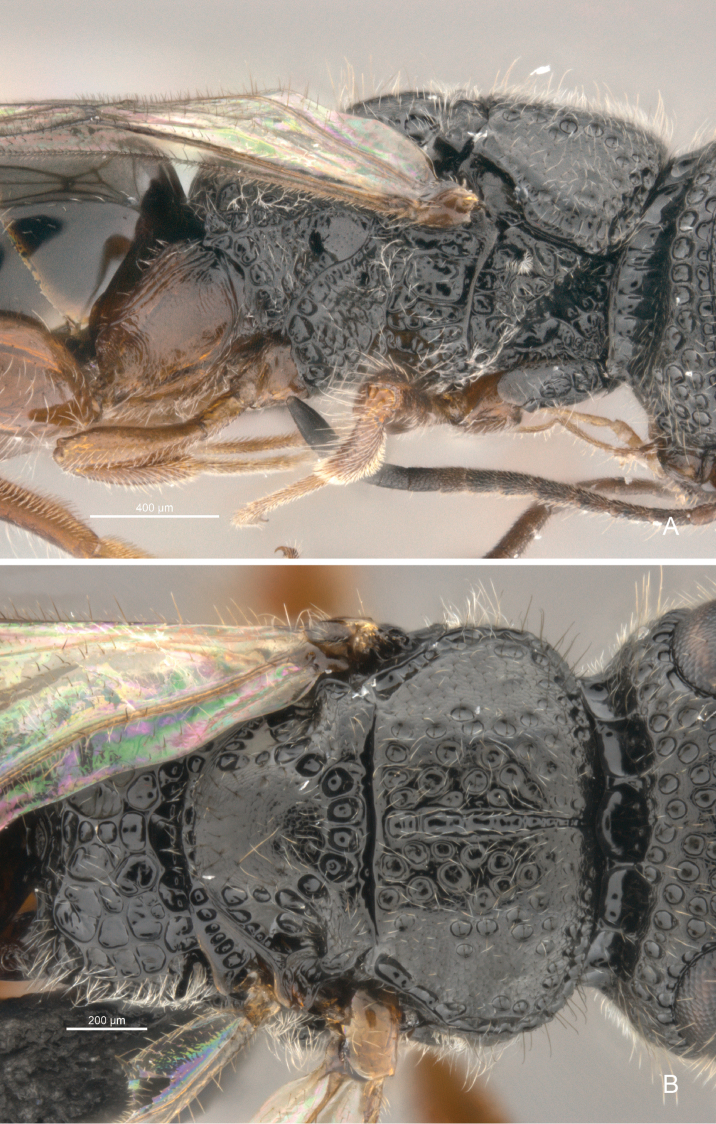
*Cryptalyra limeirai* sp. n. **A** mesosoma lateral view **B** mesosoma dorsal view. Scales bar in microns.

**Figure 9.**
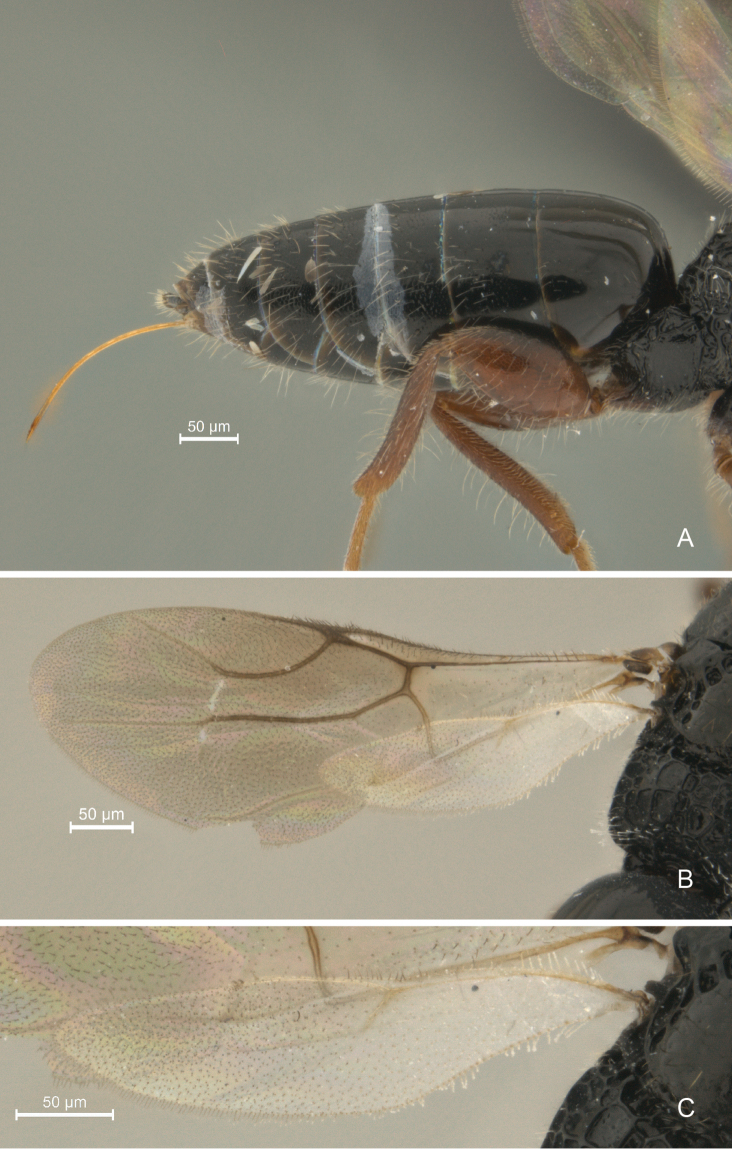
*Cryptalyra helenae* sp. n. **A** metasoma lateral view **B** forewing **C** hind wing. Scales bar in microns.

**Figure 10.**
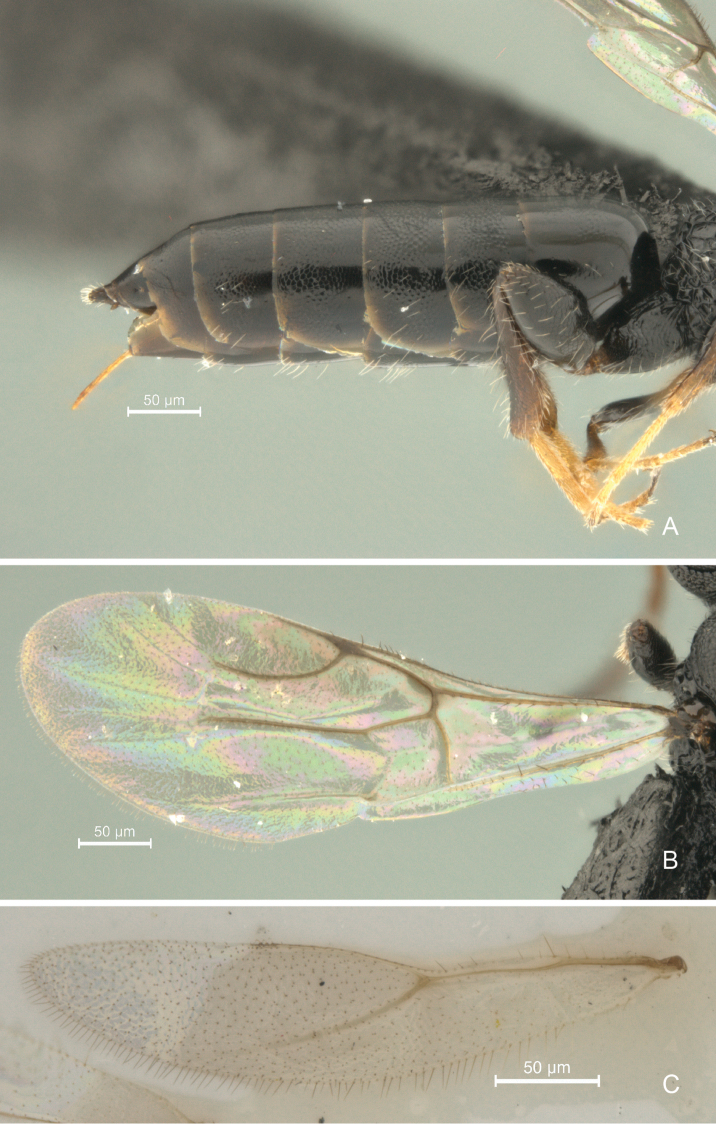
*Cryptalyra ichiroi* sp. n. **A** metasoma lateral view **B** forewing **C** hind wing. Scales bar in microns.

**Figure 11.**
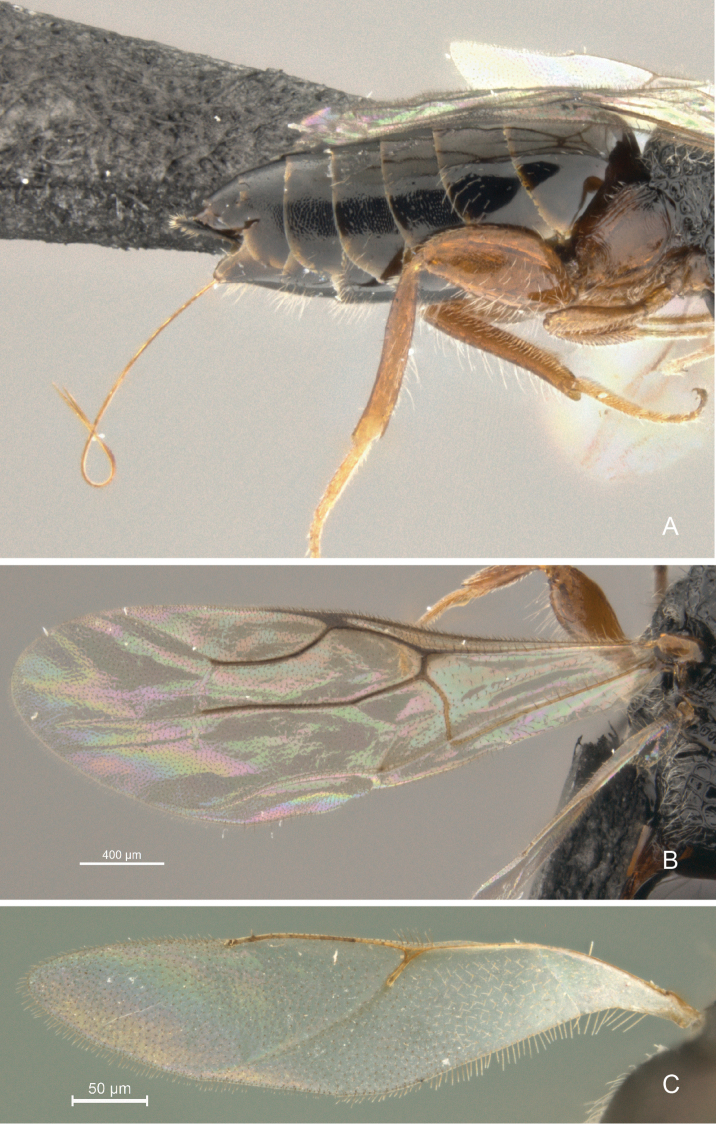
*Cryptalyra limeirai* sp. n. **A** metasoma lateral view **B** forewing **C** hind wing. Scales bar in microns.

**Figure 12.**
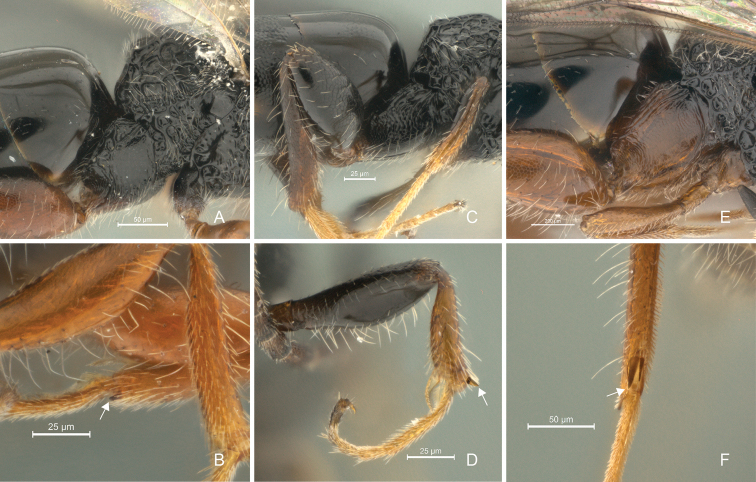
*Cryptalyra helenae* sp. n. **A** metacoxa lateral view **B** stout setae (white arrow) of mesotibia, lateral view. *Cryptalyra ichiroi* sp. n. **C** metacoxa lateral view **D** stout setae (white arrow) of fore leg, lateral view. *Cryptalyra limeirai* sp. n. **E** metacoxa lateral view **F** metatibial spurs (white srrow), posterior view. Scales bar in microns.

### Cryptalyra helenae sp. n.

Taxon classificationAnimaliaHymenopteraMegalyridae

http://zoobank.org/11EB3798-3DB0-4238-8254-E5983344B52B

Figs 1 , 4A, B , 5A, B , 6 , 9 , 12A, B

#### Material examined.

Holotype. Female. BRASIL, Maranhão, Mirador, Parque Est[adual] Mirador, Base dos Cágados, 06°48'29"S, 45°06'34"W, armadilha de Malaise, 27.ix.–02.x.2011, F. Limeira-de-Oliveira, D.W.A. Marques col[etores] (CZMA). Paratype. 1 Female, BRASIL, *Maranhão*, Carolina, PARNA Chapada das Mesas, Riacho Sucuruiu, 240m, 07°07'05.6"S, 47°18'31.6"W, armadilha de Malaise, 10–20.viii.2013, J.A. Rafael, F. Limeira-de-Oliveira, T.T.A. Silva col[etores] (CZMA).

#### Description.

**Colour.** Wings clear hyaline; tarsi and wings venation light castaneous; mandible, protibia; mesotibia, scape, pedicel, trochanters, femora, and metatibia castaneous; flagellum castaneous with four distal flagellomeres dark castaneous; head, coxae, mesosoma, and metasoma black.

**Head** (Figs 4A, B, 5A, B). Surface densely foveolate. Compound eye with short and sparse pilosity, with post-ocular sulcus compound by foveae; inner margin divergent posteriorly, in frontal view. Frons with transversal rim on anterior margin. Longitudinal sulcus extends from middle of face to vertex crest. Malar suture indistinct. Gena as long as third part width of eye, with surface uneven and densely foveolate. Occipital carina present; high, longer than half length of gena in profile; strongly scrobiculate throughout and surpassing anterior margin of pronotum laterally; occipital carina at base not curving toward mandible. Palpal formula 5:2.

**Mesosoma** (Fig. 6). Profemoral depression of pronotum conical; pronotal spiracle minute and semicircular with internal fringe of setae. Mesoscutum medially slightly convex and foveolate and lateral declined area coriaceous; median mesoscutal line (=sulcus) deep, broad and scrobiculate; notaulus absent; parapsidal signum absent; mesoscutal humeral sulcus present laterally along entire mesoscutum and scrobiculate; axillae separated medially by its own width, rounded lobe present laterally. Mesoscutellum smooth medially; scutoscutellar sulcus a row of large foveae; posterior margin of mesoscutellar disc rounded. Metanotum smooth and raised medially, scrobiculate laterally. Propodeum coarsely and irregularly foveolate to areolate; propodeal disc uneven; median longitudinal propodeal sulcus present; postero-lateral corner without tubercle.

**Metasoma** (Fig. 9A). Cylindrical, tapering posteriorly and longer than mesosoma and head in dorsal view. Abdominal tergum 2 mostly polished. Third and following abdominal tergum coriaceous with a few setae laterally. Ovipositor sheath exceedingly short; ovipositor valves entirely smooth, except four minute teeth on lower valve apically.

**Wings** (Figs 9B, C). Hind wing with Rs vein almost reaches middle of wing.

**Legs** (Figs 12A, B). Metacoxa as long as wide, with postero-dorsal surface areolate. Protibia rimmed with stout setae; metatibial setae prone to erect. Basitarsus of all legs conspicuously longer than the other tarsomeres. pro- and mesotibia with one apical spur, metatibia with two apical spurs.

#### Etymology.

The specific epithet is a patronymic honoring Helena Corte Azevedo, daughter of the third author COA.

#### Remarks.

This new species differs from *Cryptalyra colombia* in the following characters: surface of head densely foveolate; frons with transverse rim on anterior margin, and longitudinal sulcus on it extension; malar suture indistinct; occipital carina finely foveolate dorsally and ventrally; lateral parts of occipital carina curving below and nearly meeting ventrally, not curving towards mandible; mesonotum and axillae shiny but faintly shagreened and deeply pitted with numerous large round foveae, largest mesonotal foveae as broad as head foveae; median mesonotal line formed by continuous fovea, unbounded and deep; propodeum apically areolate-rugose, with longitudinal median sulcus, and posterior margin straight; metacoxa with postero-dorsal region areolate; protibia apically rimmed with stout spines; metatibia with two apical spurs; visible portion of cercus as long as 7th tergite; ovipositor with four minute teeth on lower valve apically; forewing totally hyaline; hind wing with Rs vein reaches middle of wing.

### Cryptalyra ichiroi sp. n.

Taxon classificationAnimaliaHymenopteraMegalyridae

http://zoobank.org/40AF8EB1-1E77-4244-82C2-11504351A0C2

Figs 2 , 4C, D , 5C, D , 7 , 10 , 12C, D

#### Material examined.

Holotype. Female. BRASIL, *Maranhão*, Carolina, PARNA Chapada das Mesas, Riacho Cancela, 225m, 07°06'44.2"S, 47°17'56.8"W, armadilha de Malaise, 01–15.vii.2013, J.A. Rafael, F. Limeira-de-Oliveira, T.T.A. Silva col[etores] (CZMA).

#### Description.

**Colour.** Wings clear hyaline; tarsi light castaneous; mandible, wings venation, protibia and mesotibia castaneous; flagellum castaneous with four distal flagellomeres dark castaneous; scape, pedicel, coxae, trochanters, femora, metatibia dark castaneous; head, mesosoma and metasoma black.

**Head** (Figs 4C, D, 5C, D). Surface uneven and densely coriaceous with few, sparse and unbounded foveae. Compound eye with short and sparse pilosity, without post-ocular sulcus; inner margins parallel in frontal view. Longitudinal sulcus absent on anterior part of head. Malar suture distinct. Gena as long as half width of eye, with surface uneven and densely coriaceous. Occipital carina present; low, shorter than half length of gena in profile; finely foveolate throughout and not surpassing anterior margin of pronotum laterally; occipital carina at base curving toward mandible. Palpal formula 5:2.

**Mesosoma** (Fig. 7). Pronotum with profemoral depression conical; pronotal spiracle minute and semicircular with internal fringe of setae. Mesoscutum medially slightly convex and coriaceous and lateral declined area coriaceous; median mesoscutal sulcus broad and foveolate; notaulus absent; parapsidal signum absent; mesoscutal humeral sulcus present laterally along entire mesoscutum and slightly scrobiculate; axillae separated medially by its own width, rounded lobe present laterally. Mesoscutellum smooth medially; scutoscutellar sulcus a row of small foveae; posterior margin of mesoscutellar disc sinuate. Metanotum smooth and raised medially, scrobiculate laterally. Propodeum coarsely and irregularly foveolate to areolate; area of propodeal disc in the same level; median propodeal sulcus absent; postero-lateral corner without tubercle.

**Metasoma** (Fig. 10A). Cylindrical, tapering posteriorly and longer than mesosoma and head in dorsal view. Abdominal tergum 2 mostly coriaceous with anterior area polished. Third and following abdominal tergum coriaceous with a few setae laterally. Ovipositor sheath exceedingly short; ovipositor valves entirely smooth, except five minute teeth on lower valve apically.

**Wings** (Figs 10B, C). Hind wing with Rs vein almost reaches middle of wing.

**Legs** (Figs 12C, D). Metacoxa longer than wide, with longitudinal carina dorsally. Fore- and mesotibia not rimmed with stout setae. Metatibia setae prone to erect. Basitarsus of all legs conspicuously longer than the other tarsomeres. Fore, meso- and metatibia with one apical spur, metatibial spur enclosed.

#### Etymology.

The specific epithet is a patronymic honoring Mateus Ichiro Calhau Kawada, son of the first author RK.

#### Remarks.

This new species differs from *Cryptalyra plaumanni* in the following characters: body entirely black and wings clear hyaline; eye not setose; malar suture distinct; occipital carina finely foveolate dorsally and ventrally; lateral parts of occipital carina curving below and nearly meeting ventrally, not curving towards mandible; maxillary palp 5-segmented; mesoscutum, axilla and mesoscutellar disc not punctured; pronotum, mesopleuron and metapleuron areolate; propodeum apically areolate-rugose, without distinct tubercles at postero-lateral corner; metacoxa with longitudinal carina extending midway; protibia apically rimmed with stout spines; metasoma cylindrical and coriaceous.

### Cryptalyra limeirai sp. n.

Taxon classificationAnimaliaHymenopteraMegalyridae

http://zoobank.org/1590EC1C-199A-4E17-ABAD-EE807C5DEF5D

Figs 3 , 4E, F , 5E, F , 8 , 11 , 12E, F

#### Material examined.

Holotype. Female. BRASIL, *Maranhão*, Carolina, PARNA Chapada das Mesas, Riacho Cancela, 225m, 07°06'44.2"S, 47°17'56.8"W, armadilha de Malaise, 01–15.vii.2013, J.A. Rafael, F. Limeira-de-Oliveira, T.T.A. Silva col[etores] (CZMA). Paratypes. 1 female, Mirador, Parque Est[adual] Mirador, Base da Geraldina, 419m, 06°37'25"S, 45°52'08"W, armadilha de Malaise, 01–10.x.2013, F. Limeira-de-Oliveira, L.L.M. Santos, T.L. Rocha col[etores] (CZMA); 2 females, 14–18.viii.2012, F. Limeira-de-Oliveira, J.S. Pinto, D.W.A. Marques col[etores] (CZMA); 1 female, BRASIL, *Maranhão*, Carolina, PARNA Chapada das Mesas, Riacho Corrente 288 m 07°04'24.2"S, 47°05'25.2"W, armadilha Malaise, 20–31.viii.2013, J.A. Rafael, F. Limeira-de-Oliveira, T.T.A. Silva col[etores] (CZMA); 1 female, Riacho Cancela, 225m, 07°06'44.2"S, 47°17'56.8"W, 01-15.vii.2013 (CZMA); 1 female, 01-10.x.2013 (CZMA).

#### Description.

**Colour.** Wings clear hyaline; tarsi and palpi light castaneous; coxae, trochanters, femora, and tibiae castaneous; mandible, scape, pedicel, flagellum, wing venation, and metasoma dark castaneous; head and mesosoma black.

**Head** (Figs 4E, F, 5E, F). Surface densely foveolate. Compound eye with short and dense pilosity, with post-ocular sulcus foveolate; inner margins divergent dorsally, in frontal view. Frons without transverse rim on anterior margin. Middle of face to vertex crest without longitudinal sulcus. Malar suture indistinct. Gena as long as one third of width of eye, with surface uneven and densely foveolate. Occipital carina present; high, longer than half length of gena in profile; strongly scrobiculate throughout and surpassing anterior margin of pronotum laterally; occipital carina at base not curving toward mandible. Palpal formula 5:3.

**Mesosoma** (Fig. 8). Profemoral depression of pronotum conical; pronotal spiracle minute and semicircular with internal fringe of setae. Mesoscutum medially slightly convex and foveolate and lateral declined area coriaceous; median mesoscutal line formed by continuous fovea, unbounded and widening posteriorly; notaulus absent; parapsidal signum absent; mesoscutal humeral sulcus present laterally along entire mesoscutum and scrobiculate; axillae separated medially by its own width, rounded lobe present laterally. Mesoscutellum smooth medially; scutoscutellar sulcus a row of large foveae; posterior margin of mesoscutellar disc rounded. Metanotum smooth and raised medially, scrobiculate laterally. Propodeum coarsely and irregularly foveolate to areolate; area of propodeal disc uneven; median propodeal sulcus absent; postero-lateral corner without tubercle.

**Metasoma** (Fig. 11A). Cylindrical, tapering posteriorly and longer than mesosoma and head in dorsal view. Abdominal tergum 2 mostly polished. Third and following abdominal tergum coriaceous with a few setae laterally; visible portion of cercus 2.0 × long as 7th tergite; ovipositor sheath exceedingly short; ovipositor valves entirely smooth, except six minute teeth on lower valve apically.

**Wings** (Figs 11B, C). Hind wing with Rs vein almost reaches middle of wing.

**Legs** (Figs 12E, F). Metacoxa as long as wide, with longitudinal carina dorsally. Pro- and mesotibia rimmed with stout setae; metatibia setae prone to erect. Basitarsus of all legs conspicuously longer than the other tarsomeres. Pro- and mesotibia with one apical spur, metatibia with two apical spurs.

#### Etymology.

The specific epithet is a patronymic honoring Francisco Limeira-de-Oliveira, Diptera researcher and curator of CZMA.

#### Remarks.

This new species differs from *Cryptalyra colombia* in the following characters: lateral parts of occipital carina curving below and nearly meeting ventrally, curving towards mandible; median mesoscutal line formed by continuous fovea, unbounded and widening posteriorly; propodeum coarsely and irregularly areolate; pro- and mesotibia apically rimmed with stout spines; metatibia with two apical spurs; visible portion of cercus 2.0 × long as 7th tergite; ovipositor six minute teeth on lower valve apically; forewing totally hyaline; hind wing with Rs vein reaches middle of wing.

## Discussion

Here we increased the number of described species of *Cryptalyra* from three to six. Several new character conditions broadened the genus concept: the metatibia of *Cryptalyra helenae* has two spurs in rather than one as in other species (only one spur supposed to be present in *Cryptalyra* spp., see key in Vilhelmsen et al. 2010); the head of *Cryptalyra helenae* has a longitudinal sulcus which runs medially from the middle of face to vertex crest (Figs 4A, B); the sulcus is like a line of foveae which is stronger on the vertex than on the face, this sulcus is not found in the other species. The mesonotum of *Cryptalyra limeirai* is continuous (Fig. 8B) rather than formed by foveae as in the other species such as *Cryptalyra ichiroi* (Fig. 7B). The propodeal disc of *Cryptalyra helenae* has a median longitudinal sulcus (Fig. 6B), this sulcus is not present in the other species, however *Cryptalyra depressa* presents a longitudinal wide depression characterizing this species, not being the same as found in *Cryptalyra helenae*. The dorsum of metacoxa is usually carinate (Figs 12C, E), but it is scrobiculate in *Cryptalyra helenae* (Fig. 12A). The ovipositor has usually three teeth on the dorsal side of the valve, however the *Cryptalyra helenae* ovipositor has four teeth and *Cryptalyra limeirai* and *Cryptalyra ichiroi* have six.

*Cryptalyra* was recorded from Caquetá in southern Colombia (Shaw 2003), Santa Catarina in southern Brazil (Shaw 1987) and Pará in Northern Brazil (Azevedo and Tavares 2006). These three new species here described are from Maranhão, Brazil, which is relatively close to the latter site, about 460 km in a straight line. However, the three previous nominal species were collected in rain forest and these new ones were collected in savannah, a place much drier than rain forest, although there is a regular wet season.

This genus was known only from three specimens. Now we have ten specimens, representing three times more specimens. They were collected under the scope of two projects, both coordinated by Francisco Limeira-de-Oliveira. They are “Riqueza, diversidade e composição de insetos do Parque Estadual do Mirador, Maranhão, Brasil” and “Diversidade de Diptera dos Parques Nacionais Chapada das Mesas e Serra das Confusões”. The total sampling effort was 2,700 days of Malaise trap at Parque Estadual do Mirador and 4,320 days of Malaise trap at Parque Nacional Chapada das Mesas. The traps were continuously set up in the field. Interestingly, according to Lars Vilhelmsen (pers. comm.) there are two recently collected specimens of *Cryptalyra* from French Guiana in the collection of the Natural History Museum of Copenhagen. Using the key presented herein he identified that material as apparently belonging to *Cryptalyra depressa* and *Cryptalyra ichiroi*, respectively. These specimens were also collected as a result of a continuous collecting effort, by the Société entomologique Antilles-Guyane (SEAG). As we can see, this strategy has shown to be very effective for Megalyridae. Although we have only one year of collecting in the field, continuous sampling can be useful to reveal potential seasonality. All ten specimens of *Cryptalyra* were collected from July to October.

The state of Maranhão in Brazil is here demonstrated to be a diversity spot for *Cryptalyra*. It is mostly covered by savannah, but its northwestern area is covered by Amazon rain forest, and the eastern border is covered by Caatinga vegetation. The confluence of these three main ecosystems makes Maranhão a place with huge potential for new discovery of biodiversity, especially Hymenoptera parasitoids, a group understudied in the Neotropics.

## Supplementary Material

XML Treatment for Cryptalyra helenae

XML Treatment for Cryptalyra ichiroi

XML Treatment for Cryptalyra limeirai
